# Addressing Pharmacy Admissions Declines Through a Student-Led Pre-Health Advising and Leadership System (PAALS): An Implementation Evaluation

**DOI:** 10.3390/pharmacy14010015

**Published:** 2026-01-25

**Authors:** Ashim Malhotra

**Affiliations:** Department of Pharmaceutical and Biomedical Sciences, California Northstate University College of Pharmacy, 9700 W Taron Drive, Elk Grove, CA 95758, USA; ashim.malhotra@cnsu.edu; Tel.: +1-(916)-686-8885

**Keywords:** pre-health academic advising, pharmacy admissions, Astin’s IEO theory, COEPA leadership and advocacy, pre-health career counseling, implementation science

## Abstract

To enhance PharmD student leadership and advocacy skills, combat the paucity of trained pre-health advisors for pharmacy admissions, augment community relationships, and increase pharmacy admissions volume, we designed, implemented, and assessed PAALS, a Pre-health Academic Advising and Leadership System. PAALS was grounded in Astin’s Theory of Student Involvement and evaluated using the RE-AIM implementation science framework. RE-AIM measured outcomes across Reach, Effectiveness, Adoption, Implementation, and Maintenance as indicators of PAALS’s scale, fidelity, sustainability, and institutional embedding. Analysis of PAALS using the RE-AIM framework demonstrated the following outcomes: (1) Reach: 42 P1-P3 PharmD students participated as mentors; external partnerships expanded from 2 to 8 regional high schools and community programs; and more than 25 mentored learners successfully matriculated into the PharmD program. (2) Effectiveness: students enacted sustained leadership, advocacy, and mentoring roles. (3) Adoption: voluntary uptake of mentoring and governance roles by PharmD students occurred with repeated engagement by external partner institutions. (4) Implementation: Core program components were delivered consistently using existing institutional resources. (5) Maintenance: PAALS remained operational across five academic years despite student turnover, with leadership succession and institutional embedding sustained across cohorts. Our findings demonstrate that student-led advising and advocacy ecosystems address critical gaps in pharmacy-specific pre-health advising models.

## 1. Introduction

In the United States, pre-health profession academic advising and career counseling play a critical role in guiding and preparing prospective students interested in admission to health professions programs such as MD, PharmD, DMD, BSN, and others [1]. The National Association of Advisors for the Health Professions (NAAHP) and the National Academic Advising Association (NACADA) lead the professional development of pre-health advisors, which is essential in sculpting awareness and knowledge of the pre-health professions advisors regarding health career opportunities, admissions pathways, and the competitiveness and readiness of their student advisees for these rigorous and demanding programs of study [2,3].

While a variety of resources are available for the development of pre-professional health advisors for learner preparation aimed at medical schools, Pruitt et al. and Hickey et al. noted the near vacuum of professional development resources for pre-pharmacy health advisors [4,5]. This is a predominant contributor to the rapidly waning awareness and interest in pharmacy among prospective health professions students. Furthermore, prospective students may not easily find or form a relationship with a “local champion,” such as a pharmacy student or pharmacy faculty member willing to mentor the pre-health advisee through the often confusing and multi-step admission process, including program and loan applications, PharmD program admissions essay and interview skills, and prerequisite course readiness [5].

Combined together, the trifecta of paucity of (1) pre-pharmacy advisor readiness, (2) sustained mid-to-long-term mentoring of prospective high school, community college and undergraduate students, and (3) trusted “role models,” whom applicants can turn to for help, have adversely affected pharmacy admissions, with unprecedented national decline of 49.7% between 2015–2016 and 2024–2025 [6].

To address the urgent need for reversing admission declines and the waning lack of awareness and interest in the profession of pharmacy, we created PAALS, a PharmD student-led pre-health Academic Advisors and Leadership System. The PAALS Mentoring Ecosystem includes student leadership/role handoff, pipeline advocacy, and community partnerships. PAALS’s novelty stems from preparing engaged, enthusiastic, and “local” PharmD student champions who serve as mid-to-long-term mentors, pharmacy professional advocates, and career guides for pre-health, undergraduate, and high school first-time health professions students.

Few models have operationalized sustained student involvement as both a developmental mechanism and an admissions strategy. In his seminal theory of Student Involvement [7], Astin postulated that the quality and quantity of physical and psychological energy students invest in their education (“input”) is directly proportional to their learning and personal development (“output”). While Astin’s framework has been widely employed and tested in the K12 ecosystem, its operationalization in PharmD programs has not been tested. PAALS directly tested Astin’s postulates by hypothesizing that community immersion and engagement of PharmD learners as pharmacy advocates and local champions (*the input*) would directly enhance their educational experience, engagement with the PharmD program, and reduce attrition (*the output*) by forming long-term relationships and dependencies with pre-health advisees. PAALS was designed as an operational framework that actualized leadership, self-awareness, communication, advocacy, collaboration, and problem-solving domains of the 2022 COEPA AACP report [8], providing a replicable model to link pre-health student development with the holistic, engaged, and sustained development of PharmD students.

Here we report the step-by-step protocol for constructing, implementing, and assessing PAALS premised on Astin’s Theory of Student Engagement [7] and implemented and assessed through Glasglow’s RE-AIM implementation framework [9,10]. Our data demonstrate that PAALS (1) increased PharmD students’ engagement by a threefold in observed leadership and mentoring participation roles, measured as their self-confidence and perception of engagement, (2) supported more than 25 advisees in successfully gaining admission to the PharmD program from 2020–2025, (3) increased relationships of the College of Pharmacy with local high schools from two to eight schools, and (4) had a sustained impact on PharmD students’ desire to give back to the profession and support the community even after graduation from the program. In this methods paper, we share the overall architecture, step-by-step DIY blueprint, and implementation, assessment, and transferability strategies for PAALS.

## 2. Methods

### 2.1. Study Design and Conceptual Framework

This study reports a retrospective, theory-informed implementation evaluation of a PharmD student-led pre-health mentoring and leadership ecosystem implemented at the California Northstate University College of Pharmacy (CNUCOP) between 2020 and 2025. CNUCOP offers a four-year PharmD program that is fully accredited by the Accreditation Council for Pharmacy Education (ACPE). Located across three urban campuses in the Greater Sacramento Metropolitan Region, California Northstate University (CNU) comprises six colleges: Medicine, Dental Medicine, Pharmacy, Psychology, Graduate Studies, and Health Sciences (nursing), with an overall focus on health professions education. PAALS was carefully designed as a scalable model that could be expanded from pharmacy to include other health professions students for a deeper and more meaningful impact on health professions education recruitment in this region. The project added a precedent arm to our existing and published PRIME program [11], which is a pharmacy pre-matriculation initiative started in 2018 by the author running to date, which identified the need for greater engagement and preparation of pre-health students.

PAALS was grounded conceptually in Astin’s Theory of Student Involvement [7], which posits that student learning and development are functions of the quality and quantity of physical and psychological energy students invest in educationally purposeful activities. Immediately in the wake of the COVID-19 pandemic, declines were observed in student engagement and ownership [12]. PAALS was intentionally designed to increase intensive, sustained, role-based involvement among PharmD students through authentic professional, leadership, and governance activities.

To evaluate how this theory-driven intervention was operationalized and sustained in a real-world academic setting, the study employed the RE-AIM implementation science framework consisting of the following measurable outcome stages: reach, effectiveness, adoption, implementation, and maintenance [9,10]. RE-AIM was selected to assess not whether the intervention was efficacious under controlled conditions, but whether Astin’s involvement mechanisms were successfully implemented, adopted, delivered with fidelity, and maintained within routine institutional operations.

*Intervention overview: translating Astin’s theory into practice.* The intervention consisted of two interdependent programmatic components, each designed to activate different dimensions of student involvement as described by Astin.

*Rx Mentoring Program (external involvement arm).* The Rx Mentoring Program operationalized Astin’s construct of involvement by embedding PharmD students in sustained, high-effort roles as mentors, educators, and professional advocates for pre-health learners in the local community. Rather than episodic volunteering, students engaged in repeated mentoring interactions, delivered structured workshops, reviewed applications, prepared prospective students for interviews, and represented the pharmacy profession to external audiences. These activities required substantial time investment, responsibility, and professional identity enactment, aligning with Astin’s emphasis on active, purposeful engagement.

*Faculty Class Advisor Program (internal involvement arm).* In addition to pre-matriculation mentorship, sustaining PharmD student engagement required parallel internal involvement structures, leading to the development of the Faculty Class Advisor Program. The Faculty Class Advisor Program operationalized involvement within the institution by creating formal, recurring structures for student participation in communication, governance, and continuous quality improvement. By assigning faculty advisors to each professional year cohort and embedding student representatives into standing committee workflows, the program increased students’ involvement in institutional decision-making processes.

Together, these two components were designed to increase both breadth and depth of involvement, consistent with Astin’s theory, across external professional engagement and internal institutional participation.

### 2.2. The PAALS Blueprint: Planning and Logistical Details for Reproducibility and Scalability

*Personnel involvement*: Faculty involvement included 6–8 volunteer faculty members serving as PharmD student advisors, mentors, or institutional liaisons, with recurring engagement through structured meetings, outreach events, and admissions-related activities. PAALS was coordinated by the Assistant Dean of Accreditation, who oversaw both the Rx Mentoring Program and the Faculty Class Advisor Program, including logistics, operations, and addressing emergent PharmD student and CNUCOP faculty concerns. This individual contributed 10%-time effort towards PAALS and was assisted by the Assistant Dean of Student Affairs and Admissions (2%-time allocation), the Admissions Director (1% time effort), the faculty leading the Faculty Class Advisor program (3 h per semester), and the administrative staff (1 h per week for 4 weeks per semester).

*Strategy for Building Relationships with the School District and Embedding PharmD students in service learning.* The Rx Mentoring arm was coordinated by a PharmD student volunteer who was trained by the Assistant Dean of Accreditation regarding the goals, objectives, and deliverables of PAALS (2-h introductory session). The PharmD student lead subsequently trained the 42 other PharmD student volunteers, thereby validating the train-the-trainer part of our sustained leadership approach. The student lead also drafted a written proposal detailing (1) the steps for mentoring, (2) advising protocols for prospective students, (3) protocols for working with minor high school students and high school faculty advisors, (4) information about PharmD pre-requisite courses, (5) information about the community colleges and four-year programs in our vicinity where these courses could be completed, and (6) resources for CNUCOP admissions preparation. The Assistant Dean for Accreditation identified and developed relationships with local high school teachers, principals, and administrators of the Elk Grove School District (EGSD) in Northern California. The student leader and the volunteers officially joined existing EGSD “mentor” programs. The Assistant Dean served on the board of the largest high school that had the signature EGSD mentor program, gradually expanding health profession student mentorship from pharmacy to other colleges within our system, including Medicine, Dental Medicine, and Nursing.

*The Purpose and Logistics of the Faculty Class Advisor Program*. Monthly cohort meetings and structured feedback loops required students to invest cognitive and emotional energy in understanding institutional systems, articulating peer concerns, and engaging constructively with administration.

### 2.3. Assessment Strategy

**Figure 1:**
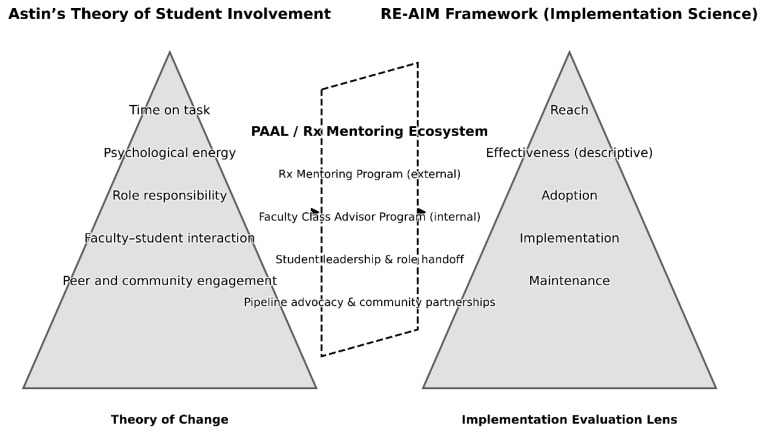
Conceptual Integration of Astin’s Theory of Student Involvement (**left**) and the RE-AIM Implementation framework (**right**), Operationalized through PAALS (**center**).

Astin’s theory informed the design of the intervention, while implementation science frameworks were used to evaluate how that theory was operationalized in practice. This novel assessment approach enshrines sustainable change and is explained in Figure 1.

Consistent with Astin’s theory, student engagement was operationalized behaviorally rather than psychometrically. Engagement was assessed through documented participation in sustained mentoring roles, leadership responsibilities, committee involvement, and longitudinal continuity of involvement across academic years, rather than through self-report engagement scales.

### 2.4. Implementation and Data Sources

RE-AIM was selected because it prioritizes sustainability, scalability, and institutional embedding. Using the RE-AIM framework, the evaluation examined five implementation domains: reach, effectiveness (descriptive), adoption, implementation, and maintenance [9,10]. Data sources included program proposals, mentor rosters, faculty advisor documentation, outreach materials, presentation slides, administrative records, email correspondence, and longitudinal AACP Graduating Student Survey data (2022–2025).

Given the implementation-focused nature of the study, outcomes were analyzed descriptively. Counts, longitudinal trends, and categorical indicators were used to characterize implementation progress across RE-AIM domains rather than inferential hypothesis testing. Outcomes were analyzed descriptively and mapped to RE-AIM domains, with explicit attention to how observed outcomes reflected increased student involvement consistent with Astin’s theoretical propositions. The interrelationships between Astin’s IEO theory, RE-AIM framework, and the implementation measures utilized for evaluating the effectiveness of PAALS are detailed in Table 1, with specific examples for each category.

In addition to administrative counts and program documentation, qualitative evidence of impact was drawn from contemporaneous student reflections, unsolicited written feedback, and post-participation communications (e.g., emails, proposals, leadership transition documents). These materials were not subjected to formal qualitative coding but were used to triangulate observed engagement, leadership enactment, and professional identity development, consistent with Astin’s emphasis on behavioral and psychological investment.

Document consistency through the years was ensured by the structural approach of having the Assistant Dean of Accreditation and his office lead the project through a 10% time allocation effort. Thus, all instructional and guidance documents, outcome reports, flyers, internal audits, frequency of mentoring sessions, and additional materials used by PharmD students for mentoring were stored in password-protected files in this office.

## 3. Results

The Results Section below is presented in alignment with the approach of an implementation science philosophy following the RE-AIM model, with the following outcome sections: Reach, Effectiveness, Adoption, Implementation, and Maintenance. The PAALS mentoring ecosystem incorporated structured student leadership succession, admissions pipeline advocacy activities, and sustained community partnerships, each of which was explicitly operationalized and tracked across implementation years.

All outcomes are reported descriptively to characterize the implementation trajectory rather than to estimate causal effects.

Table 2 summarizes descriptive implementation indicators across RE-AIM domains using counts, frequencies, and duration of engagement rather than inferential estimates, consistent with implementation evaluation methodology.

**Figure 2:**
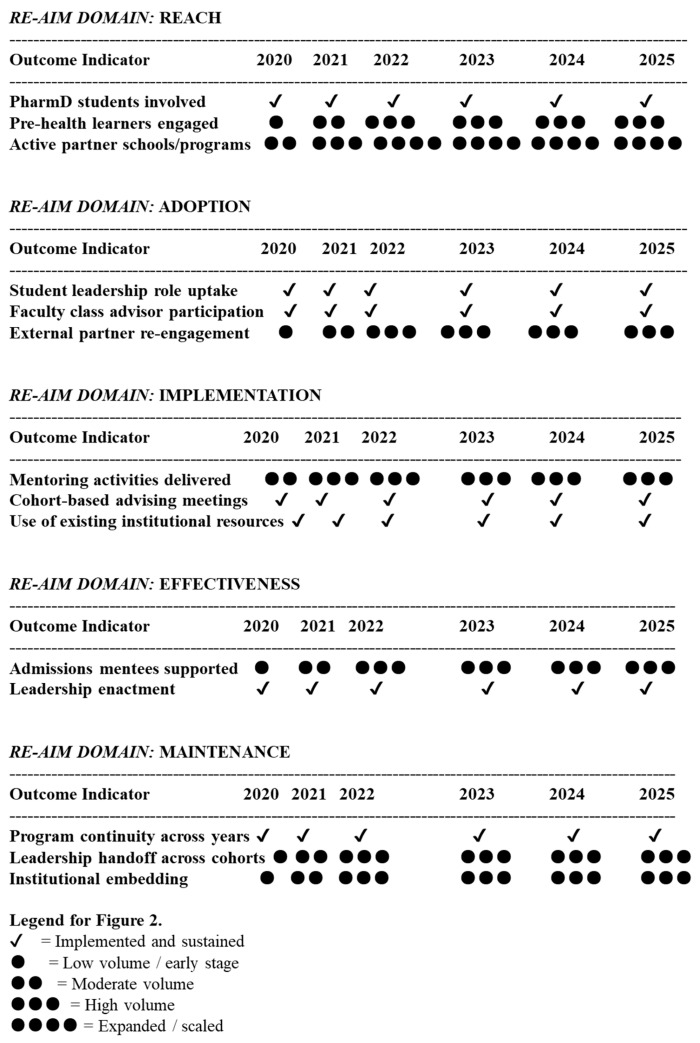
Longitudinal Implementation Outcomes by RE-AIM Domain (2020–2025).

Figure 2 summarizes PAALS outcomes for each of the RE-AIM framework’s implementation categories, especially aligned to the temporal development of the project. Figure 2 provides a longitudinal, descriptive visualization of PAALS implementation across the five RE-AIM domains (Reach, Effectiveness, Adoption, Implementation, and Maintenance) from 2020 to 2025. Symbols reflect relative levels of implementation activity over time rather than inferential effect sizes. Check marks (✔) indicate sustained implementation of a core component, while increasing numbers of circular markers (● to ●●●●) denote progression from early to expanded levels of activity, as defined in the figure legend. The figure is intended to illustrate implementation scope, durability, and maturation across domains rather than causal effectiveness.

### 3.1. RE-AIM Domain: Reach

The intervention achieved substantial reach across both student and community populations. In the initial year of implementation (2020–2021), 42 PharmD students formally participated as mentors in the Rx Mentoring Program, representing multiple professional year cohorts (P1–P3s). Externally, the mentoring program expanded its reach from partnerships with two local high schools to approximately eight schools over the study period, increasing exposure of underserved pre-health learners to pharmacy education and careers. Internally, the Faculty Class Advisor Program reached all enrolled PharmD students through cohort-based meetings spanning the P1–P4 years, ensuring universal access to involvement opportunities within the institution. From an Astin model perspective, this reach reflects the availability of involvement opportunities, a necessary precondition for student development.

### 3.2. RE-AIM Domain: Effectiveness

Although causal effectiveness was not assessed, several outcomes consistent with increased student involvement were observed. Over the 2020–2025 period, more than 25 individuals who engaged with the Rx Mentoring ecosystem were supported in successfully gaining admission to the PharmD program, suggesting alignment between sustained mentoring involvement and admissions pipeline goals.

More importantly for Astin’s theory, PharmD students participating in the intervention assumed roles that required meaningful investment of time, responsibility, and professional identity enactment. Students functioned as mentors, workshop leaders, admissions ambassadors, and committee representatives, engaging in activities that demanded preparation, communication, teamwork, and advocacy. These role-based behaviors represent the kind of high-intensity involvement Astin identifies as most strongly associated with developmental gains.

At the institutional level, longitudinal AACP Graduating Student Survey data from 2022 to 2025 demonstrated stable or improving trends in domains related to teamwork, professional preparation, and engagement. While not designed to evaluate the intervention directly, these trends provide contextual evidence of a supportive environment during the period in which involvement-focused structures were implemented.

### 3.3. RE-AIM Domain: Adoption

Adoption of the intervention was observed across students, faculty, and external partners. PharmD students voluntarily adopted mentoring and governance roles beyond curricular requirements, indicating perceived value and alignment with professional goals. Faculty adopted advisor roles with recurring responsibilities, integrating the program into routine academic operations. External partners, including high schools and district-affiliated programs, actively engaged with the mentoring model and requested continued collaboration. This multi-level adoption suggests that the intervention successfully translated Astin’s involvement principles into practices that stakeholders were willing to embrace.

### 3.4. RE-AIM Domain: Implementation

Core components of both program arms were implemented with fidelity to their original design. The Rx Mentoring Program consistently emphasized sustained, structured engagement rather than one-time outreach events, maintaining alignment with Astin’s emphasis on duration and intensity of involvement. The Faculty Class Advisor Program preserved a regular meeting cadence, incorporated student committee reporting, and employed documented feedback loops connecting student input to institutional decision-making. Importantly, implementation was feasible using existing institutional resources, demonstrating that high-involvement structures can be embedded without excessive financial or administrative burden.

### 3.5. RE-AIM Domain: Maintenance

The intervention demonstrated maintenance across multiple academic years despite student turnover and evolving institutional contexts. Both program components persisted beyond initial launch and adapted over time without loss of core involvement mechanisms. Continued student participation, faculty engagement, and external partner interest indicate that the intervention became embedded within institutional routines. From an Astin perspective, this maintenance reflects the institutionalization of involvement opportunities, increasing the likelihood that developmental benefits could be sustained across cohorts.

### 3.6. Engagement and Leadership Impact on PharmD Student Participants

Across the 2020–2025 implementation period, PAALS engaged approximately 42 PharmD student mentors, spanning P1–P4 cohorts, who collectively contributed an estimated 1200–1500 volunteer hours in advising sessions, workshops, application reviews, and outreach activities. Participation was longitudinal rather than episodic, with several students serving in leadership roles for two or more academic years and facilitating formal leadership handoff to subsequent cohorts. This is a viable implementation and feasibility outcome because literature documents that student engagement during and in the aftermath of the COVID-19 pandemic and with the emergent digital technologies has waned [12].

Qualitative student feedback consistently reflected increased confidence, professional ownership, and leadership skill development. Representative comments included statements such as: “This was the first time I felt like I was representing the profession, not just preparing to enter it,” and “Mentoring pre-health students forced me to articulate why pharmacy matters and helped clarify my own professional goals.”

These observations align with Astin’s constructs of involvement through sustained time investment, role responsibility, and psychological commitment, providing evidence of impact beyond simple participation counts.

## 4. Discussion

### 4.1. Integrating Student Development with Admissions Pipeline Outcomes

Admissions outreach in pharmacy education is frequently siloed from student development initiatives, limiting opportunities for meaningful student engagement and professional identity formation [1,10]. PAALS intentionally integrated these functions. Within the PAALS mentoring ecosystem, student leadership role handoff, admissions pipeline advocacy, and community partnerships were treated as core implementation components and were systematically documented as part of the RE-AIM domains of adoption, implementation, and maintenance.

Between 2020 and 2025, more than 25 pre-health learners engaged through PAALS were supported in gaining admission to the PharmD program (Figure 2, Effectiveness domain). While causality cannot be inferred, these outcomes coincide with sustained student mentoring involvement and expanded community reach. Simultaneously, PharmD students participating in PAALS engaged in repeated articulation of pharmacy career pathways, admissions requirements, essay coaching, and interview preparation. These activities demanded advanced communication, leadership, and advocacy skills and were sustained across cohorts, as evidenced by consistent leadership enactment and program maintenance (Figure 2, Effectiveness and Maintenance domains). By linking admissions pipeline development with student learning outcomes, PAALS addresses a literature gap in integrated educational–recruitment models.

### 4.2. PAALS Operationalizes Leadership and Advocacy Through Sustained Student Involvement

A persistent gap in PharmD education is the lack of scalable, assessable mechanisms for achieving leadership and advocacy outcomes emphasized in the COEPA domains [8,11,12,13,14] and the 2025 ACPE Standards [15]. These competencies are often addressed through episodic activities that are difficult to sustain or evaluate longitudinally [14,16]. As shown in Table 3, PAALS addressed this gap by embedding leadership and advocacy within sustained, role-based student involvement.

As shown in Figure 2 (Adoption and Implementation domains), PharmD students consistently assumed formal leadership roles across all years of implementation (2020–2025), with uninterrupted student leadership uptake and faculty advisor participation. Activities, including mentoring, admissions outreach, workshop facilitation, and governance engagement, required sustained time commitment and responsibility from CNUCOP PharmD student champions, aligning directly with Astin’s constructs of *time on task* and *psychological energy*.

Table 1 maps these activities explicitly to Astin’s framework and RE-AIM domains, demonstrating how leadership and advocacy were operationalized with fidelity and maintained over time. By transforming leadership from a discrete outcome into a continuous practice, PAALS closes a long-standing gap between accreditation expectations and measurable student engagement.

### 4.3. Addressing Deficits in Pre-Pharmacy Advising Through a Student-Led Model

The national decline in PharmD applications [6] has been accompanied by diminishing availability of pharmacy-specific pre-health advising [4]. Unlike medicine or dentistry, few trained advisors specialize in pharmacy pathways, leaving prospective students without informed guidance.

PAALS mitigated this structural deficit by positioning PharmD students and faculty as “local champions.” External reach expanded from two to approximately eight partner high schools over the implementation period (Figure 2, Reach domain), while internal reach included all enrolled PharmD cohorts (P1–P3) annually. This redistributed pre-health PRESSER advising responsibilities in a cost-conscious manner while enhancing PharmD student engagement. By leveraging student expertise supported by faculty oversight, PAALS operationalized Astin’s involvement principles while addressing a documented gap in pharmacy admissions advising.

### 4.4. Sustainability and Transferability Through Implementation Science

Many educational innovations lack evidence of sustainability beyond initial launch [17]. Using the RE-AIM framework, PAALS demonstrated sustained implementation across all five domains. Figure 2 shows continuous program operation, leadership handoff across cohorts, and institutional embedding from 2020 to 2025. Core components were delivered with fidelity using existing resources, without external funding or additional staffing.

By explicitly linking Astin’s student involvement theory with RE-AIM implementation outcomes (Table 1), this study addresses a gap in pharmacy education literature where theory-driven interventions are rarely evaluated through implementation science. PAALS offers a reproducible blueprint for institutions seeking sustainable, accreditation-aligned student engagement systems.

### 4.5. Contribution to Literature

PAALS demonstrates that sustained, theory-informed student involvement can simultaneously advance leadership development, admissions pipeline strength, and institutional engagement. By documenting multi-year reach, adoption, implementation fidelity, and maintenance, this study closes the gap between aspirational educational standards and operationally measurable practice in PharmD education. These skills are not only important to co-curricular and holistic development of students, but also form the basis of teamwork, which is essential to practice-ready interprofessional teams, the emerging frontier of healthcare in the U.S. [18].

## 5. Limitations

This study’s primary limitation was PAALS implementation and evaluated at a single institution using a retrospective, descriptive implementation design. Outcomes such as admissions pipeline success, leadership enactment, and student engagement were assessed through observable participation, institutional records, and longitudinal implementation indicators. However, this limitation is consistent with the study’s purpose: to evaluate *real-world operationalization*, *sustainability*, *and transferability* of a theory-informed educational system using the RE-AIM framework rather than to test efficacy under experimental conditions.

## 6. Conclusions

PAALS demonstrates that a theory-informed, student-led mentoring and leadership system can be sustainably embedded within a PharmD program to advance leadership, advocacy, and admissions pipeline outcomes while maintaining high levels of student engagement. By operationalizing Astin’s Theory of Student Involvement through a RE-AIM–guided implementation framework, PAALS offers a scalable and transferable model for addressing workforce awareness and enrollment challenges in pharmacy education, which have been challenging to address through program-wide strategies [19].

## Figures and Tables

**Table 1:** Conceptual Alignment of Astin’s IEO Theory and the RE-AIM Implementation Framework within the PAAL Network.

Framework Domain	Theoretical Construct	Programmatic Operationalization (PAAL Network)	Assessment Evidence
Astin IEO Theory	*Time on task*	Sustained student participation in mentoring, outreach, and advising activities	Documented longitudinal student participation across academic years
Astin IEO Theory	*Psychological energy*	Student ownership of mentoring design, outreach planning, and peer leadership roles	Student-authored proposals, presentations, and leadership continuity
Astin IEO Theory	*Faculty–student interaction*	Faculty Class Advisor Program and co-led mentoring initiatives	Structured advisor assignments and recurring faculty–student engagement
RE-AIM Framework	*Reach*	Engagement of PharmD students, faculty advisors, and external partner schools	Growth in number and diversity of participants and partner institutions
RE-AIM Framework	*Adoption*	Voluntary uptake of mentoring and advising roles by students and faculty	Repeated program participation and leadership succession
RE-AIM Framework	*Implementation*	Standardized mentoring structures and advising workflows	Consistent execution across cohorts and academic years
RE-AIM Framework	*Maintenance*	Continuation of programs beyond initial implementation period	Sustained operation across multiple years

The phrases in italics are operational phrases that show how each stage of Astin’s IEO model was actualized.

**Table 2:** Descriptive Implementation Indicators of PAALS by RE-AIM Domain (2020–2025).

RE-AIM Domain	Descriptive Implementation Indicators
Reach	PharmD student mentors: *n* = 42 (P1–P4) Pre-health learners matriculated into PharmD: >25 Partner schools/programs: 2 → ~8 PharmD cohorts reached via Faculty Class Advisor Program: All cohorts annually
Effectiveness (Descriptive)	Cumulative student volunteer effort: ~1200–1500 h Mentoring engagement duration: multi-year (up to 4 years per student) Activity types delivered: mentoring, workshops, admissions advocacy, committee representation
Adoption	Faculty advisors participating: ~6–8 Student leadership uptake: annual, voluntary External partner re-engagement: recurring across years
Implementation	Cohort advising meetings: monthly Mentoring model: longitudinal, train-the-trainer Resources used: existing institutional infrastructure only
Maintenance	Program duration: 5 academic years Leadership handoffs: annual, cohort-based External funding required: none

**Table 3:** Mapping PAALS Outcomes to COEPA 2022 Leadership and Advocacy Domains.

COEPA Domain	COEPA Outcome Description	PAALS Outcome Evidence from This Study
**Leader** *Domain 2.9*	Demonstrate the ability to influence, guide, and support achievement of shared goals within teams and organizations	PharmD students served as sustained mentors, outreach leaders, admissions ambassadors, and committee representatives, requiring leadership, coordination, and responsibility.
**Advocate** *Domain 2.5*	Promote the profession and advance its value to stakeholders and the public	Students acted as pharmacy career advocates through structured mentoring, workshops, and outreach to high school, community college, and undergraduate pre-health learners.
**Communicator** *Domain 2.2*	Communicate effectively with diverse audiences using appropriate strategies	Students delivered workshops, interview preparation sessions, application guidance, and advising interactions with prospective applicants and institutional partners.
**Collaborator** *Domain 2.7*	Engage in team-based activities demonstrating shared responsibility and respect	Mentoring and Faculty Class Advisor activities required collaboration with faculty, peers, admissions staff, and external educational partners.
**Professional** *Domain 3.2*	Demonstrate accountability, ethical behavior, and commitment to professional standards	Longitudinal engagement, leadership continuity across cohorts, and sustained community involvement reflected professional responsibility and service.
**Self-Aware** *Domain 3.1*	Reflect on personal development, professional identity, and role within the profession	High-intensity mentoring and leadership roles required students to enact, reflect upon, and refine their emerging professional identity as pharmacists.

Terms in bold are COEPA domains, with the Domain numbers illustrated in italics.

## Data Availability

The original contributions presented in this study are included in the article Further inquiries can be directed to the author.

## References

[1] Rios-Fetchko F., Carson M., Tapia M., Fernandez A., Coffman J. (2024). Disparities in pre-health advising across California’s public universities. PLoS ONE.

[2] McGill C.M. (2019). The professionalization of academic advising: A structured literature review. NACADA J..

[3] National Association of Advisors for the Health Professions (2017). Health Professions Admissions Guide: Strategy for Success.

[4] Pruitt S., Darley A., Dennison E. (2023). Increasing the PharmD Pipeline, Encouraging Student Success, and Supporting the Underserved Through Pre-Pharmacy Advising. Am. J. Pharm. Educ..

[5] Hickey E., DiPiro J., Romanelli F. (2019). Prospective health professions students’ misperceptions about pharmacists. Am. J. Pharm. Educ..

[6] American Association of Colleges of Pharmacy (2025). 2024–2025 PharmCAS Applicant Data Report.

[7] Astin A.W. (1984). Student involvement: A developmental theory for higher education. J. Coll. Stud. Pers..

[8] Medina M.S., Farland M.Z., Conry J.M., Culhane N., Kennedy D.R., Lockman K., Malcom D.R., Mirzaian E., Vyas D., Steinkopf M. (2023). The AACP Academic Affairs Committee’s Guidance for Use of the Curricular Outcomes and Entrustable Professional Activities (COEPA) for Pharmacy Graduates. Am. J. Pharm. Educ..

[9] Glasgow R.E., Vogt T.M., Boles S.M. (1999). Evaluating the public health impact of health promotion interventions: The RE-AIM framework. Am. J. Public Health.

[10] Glasgow R.E., Harden S.M., Gaglio B., Rabin B., Smith M.L., Porter G.C., Ory M.G., Estabrooks P.A. (2019). RE-AIM Planning and Evaluation Framework: Adapting to New Science and Practice With a 20-Year Review. Front. Public Health.

[11] Malhotra A., Kreys E., Feng X. (2022). Impact of a prepharmacy program on students’ self-awareness of pharmacist professional identity: Comparison between virtual and in-person settings. Pharmacy.

[12] Courtney J., Titus-Lay E., Malhotra A., Nehira J., Mohamed I., Mente W., Le U., Buckley L., Feng X., Vinall R. (2022). COVID-19-Driven Improvements and Innovations in Pharmacy Education: A Scoping Review. Pharmacy.

[13] Gortney J.S., Lacroix M., Dunn B.L. (2025). Fostering Advocacy in Student Pharmacists: Strategies for Curricular Integration and Assessment. Am. J. Pharm. Educ..

[14] Janke K.K., Bechtol R.A., Smith K.J. (2025). Shifting the Leadership Assessment Paradigm in Doctor of Pharmacy Curricula. Am. J. Pharm. Educ..

[15] Accreditation Council for Pharmacy Education (2024). Accreditation Standards and Key Elements for the Professional Program in Pharmacy Leading to the Doctor of Pharmacy Degree (“Standards 2025”).

[16] Fjortoft N. (2016). The Challenge of the Accreditation Council for Pharmacy Education’s Standard Four: Identifying, Teaching, Measuring. Am. J. Pharm. Educ..

[17] Wiltsey Stirman S., Kimberly J., Cook N., Calloway A., Castro F., Charns M. (2012). The sustainability of new programs and innovations: A review of the empirical literature and recommendations for future research. Implement. Sci..

[18] Malhotra A., Yang C., Feng X. (2022). Application of constructivism and cognitive flexibility theory to build a Comprehensive, Integrated, Multimodal Interprofessional Education and Practice (CIM-IPEP) program. J. Interprof. Care.

[19] Wong W.J., Lee R.F.S., Chong L.Y., Lee S.W.H., Lau W.M. (2023). Work readiness of pharmacy graduates: An exploratory study. Explor. Res. Clin. Soc. Pharm..

